# Use of Recombinant CP2 and CP23 Antigens of *Cryptosporidium parvum* for Serodiagnosis of Human Cryptosporidiosis

**DOI:** 10.52547/ibj.3801

**Published:** 2022-10-29

**Authors:** Gholamreza Barzegar, Ehsan Ahmadpour, Bahador Shahriari, Rahmat Solgi, Mohammad Hossein Motazedian

**Affiliations:** 1 Department of Parasitology and Mycology, School of Medicine, Shiraz University of Medical Sciences, Shiraz, Iran;; 2 Infectious and Tropical Diseases Research Center, Tabriz University of Medical Sciences, Tabriz, Iran;; 3 Department of Parasitology and Mycology, School of Medicine, Shiraz University of Medical Sciences, Shiraz, Iran;; 4 Cellular and Molecular Research Center, Birjand University of Medical Sciences, Birjand, Iran

**Keywords:** Cryptosporidiosis, Enzyme-Linked Immunosorbent Assay, Western blotting

## Abstract

**Background:**

*Cryptosporidium* *parvum* is an important coccidian parasite infecting many mammals, including human. This parasite can manifest as chronic severe diarrhea in immunocompromised individuals, especially those with AIDS. The present study reports the recombinant production of rP2 and rP23 antigens of *C. parvum* as antigens for detecting human cryptosporidiosis using indirect ELISA tests.

**Methods:**

The coding sequences of rP2 and rP23 proteins were codon-optimized, commercially synthesized and sub-cloned in the pET28a expression vector. The expressed proteins were purified by Ni-NTA column chromatography and confirmed by Western blotting. The efficacy of rP2/rP23 proteins for serodiagnosis was evaluated by positive (n = 20) and negative (n = 20) human sera, confirmed by the Ziehl-Neelsen staining as the gold standard test.

**Results:**

In ELISA test, the sera from *C. parvum*-infected patients reacted strongly to rP2/rP23. The sensitivity and specificity related to the diagnostic potential of rP2/rP23 in the ELISA assay were 100%.

**Conclusion:**

Our results showed that combination of rP23 and rP2 antigens in ELISA significantly increases the performance of *C. parvum *serodiagnosis in human cryptosporidiosis.

## INTRODUCTION

*Cryptosporidium parvum *is a coccidian parasite recognized as a major cause of human diarrheal disease, namely cryptosporidiosis. This parasite can infect intestinal epithelial cells and is transmitted via orofecal route^[^^1^^]^. 

Cryptosporidiosis is associated with asymptomatic or self-limited diarrhea in immunocompetent people and acute diarrhea with weight loss in immunocompromised individuals, particularly those with AIDS^[^^2^^]^. The fecal smear preparation, Ziehl-Neelsen staining, and microscopic observation are routinely used to diagnose cryptosporidiosis; however, these methods have been proved to be ineffectual due to low sensitivity and specificity^[^^3^^]^. Therefore, molecular tests have been employed for final confirmation of the diagnosis^[^^4^^]^, though it needs expertise and specialized equipment^[^^4^^,^^5^^]^. 

In recent years, many studies have utilized crude and recombinant antigens for diagnosing crypto-sporidiosis^[^^3^^,^^7^^]^. The coproantigens of *C. parvum* have been used for detection of the infection with relatively acceptable sensitivity and specificity^[^^6^^]^. The ELISA was performed by crude *C. parvum* antigen with low specificity. However, today, different recombinant *Cryptosporidium* antigens with high specificity have been introduced^[^^8^^]^. The P23 and P2 proteins of *Cryptosporidium* are strongly immunogenic in inducing anti-P23/P2 antibody responses in the early stages of infection. Thus, these proteins can serve as an important candidate antigen for vaccine and serodiagnosis of cryptosporidiosis^[^^9^^]^. 

Previously, rP23 and rP2 antigens have been employed for serodiagnosis of cryptosporidiosis in animals, particularly cattle and calves^[^^10^^-^^12^^]^. However, a subsequent study has employed the two mentioned antigens for detecting the anti-*C. parvum *antibodies in human samples^[7]^. In the current study, the rP23 and rP2 were utilized simultaneously in the indirect ELISA method for detecting anti-*Cryptosporidium* antibodies in human samples. 

## MATERIALS AND METHODS

**Human** **serum samples**

Human sera were collected from *C. parvum*-infected patients (n = 20) and healthy individuals (n = 20) and confirmed by the Ziehl-Neelsen method. The sera were provided by Tabriz Medical School, Tabriz, Iran, and stored at -20 °C until testing.

**Amplification of** ***P2*** **and** ***P23 *genes**

The sucrose gradient method was used to isolate the *Cryptosporidium* oocysts from the feces of HIV-positive patients, as described previously^[^^13^^]^. The oocysts were prepared and stained using the modified Ziehl-Neelsen method^[^^14^^]^. DNA was extracted by a DNA extraction kit (Favorgen Biotech, Taiwan) following the instructions provided by the company. The* C. parvum* genome was confirmed by specific primers, and both *P23* (accession number: U34390) and *P2* (accession number: AF099744) genes were amplified using the same primers (Table 1). The amplified sequences of the two genes were cloned separately in a pGEM-T plasmid. The *P2*-pGEM-T and *P23*-pGEM-T were transformed into *E. coli* TOP 10 and then confirmed by DNA sequencing^[^^15^^]^. 

**Expression of recombinant P2 and P23** **proteins**

The *P2* and *P23* sequences were codon optimized, chemically synthesized and directly inserted into the expression vector pET-28a(+) (Novagen, Singapore) at the specified restriction site (General Biosystem, USA). For the affinity purification of recombinant proteins, a 6×His-tag coding sequence was inserted to the upstream of the stop codon of the synthetic gene. The rP2-pET-28a and rP23-pET-28a were transformed separately into *E. coli* BL21 (DE3) as a competent cell (Thermo Fisher, USA(. *E. coli BL21* containing rP2/23-pET-28a was cultured in a 5-ml broth medium containing 50 mg/L of kanamycin. When the OD at 600 nm reached 0.6, the protein expression was induced by adding Isopropyl β-D-1-thiogalactopyranoside (IPTG) to the LB broth medium (1 mM) at 30 °C for 24 h. Then rP2/23- pET-28a cloned genes were confirmed by direct DNA sequencing.

**Protein purification and Western blot analysis**

Expression of the rP23/rP2 protein was analyzed on a 12% SDS-PAGE gel. The recombinant proteins containing His-Tag in their N-terminal were purified by a column containing 5 ml of Ni2^+^-agarose gelatin. For final purification, a phosphate buffer dialysis bag containing saline, pH 7.4, was used. The Bradford method was applied to measure the final concentration of recombinant proteins. For determination of immunoreactivity, the purified rP23/rP2 protein was initially separated by SDS-PAGE (12%) and then transferred to a 0.2-mm polyvinylidene difluoride membrane (Sartorius, Germany). Protein contents of rP23/rP2 antigens in *E*. *coli* were compared with those of *E. coli* strain BL21 and pET-28a without insertion. The polyvinylidene difluoride membrane was then blocked with 4% BSA solution at 4 °C for 1.5 h. *C. parvum*-infected and uninfected human sera were used as primary antibodies in blocking buffer (Tris-buffered saline containing 0.1% [v/v] Tween-20 [TBST] and BSA [2%]). The goat anti-human IgG HRP (+) antibody (Thermo Fisher) was used as a secondary antibody. The bands in Western blotting were visualized using diaminobenzidine (TIANGEN, Beijing, China), as the substrate.

**Table 1:** Primers used in this study

**Gene**	**Accession no.**	**Primer**	**Sequence**
*18S r RNA*	AF093489	F	5’-AAGCTCGTAGTTGGATTTCTG-3’
	AF093489	R	5’-TAAGGAACAACCTCCAATCTC-3’
			
*P23*	U34390	F	5’-ACGGATCCAAAAATGGGTTGTT-3’
	U34390	R	5’- ACCTCGAGTAATTTAGGCATCA-3’
			
*P2*	AF099744	F	5’-GGGGATCCCCCTGGTTCCGCGTGGATCCATGGGT-3’
	AF099744	R	5’-CGCCCCTCGAGATTTAATTAGTCA AAC-3’

**ELISA assay**

**Fig. 1:**
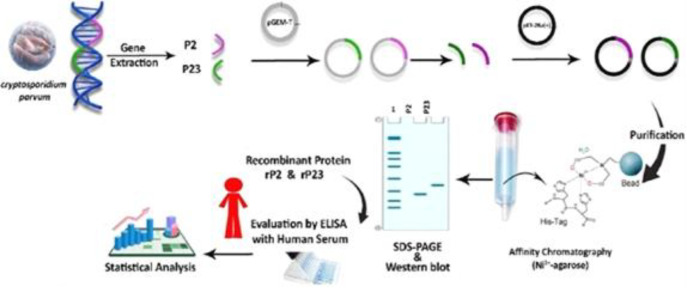
Schematic overview of this study

Performance of rP23/rP2 ELISA was evaluated by the sera of the infected individuals after checkerboard titration. Flat-bottom 96-well ELISA plates were coated with 2.5, 5, 10, 20, and 40 μg/ml of rP2 and rP23 in PBS-T using 100 μl per well and incubated at 4 °C overnight. The plates were blocked with BSA (5%) at 37 °C for 2 h. Individual serum samples were diluted 1:10, 1:100, 1:1000, 1:10000 in 0.05% Tween 20-PBS, applied to the wells in duplicate and incubated at 37 °C for 2 h. After washing the plates, bounded antibodies were detected by incubation with conjugated goat anti-human IgG (Thermo Fisher) at a 1:1000 dilution in PBS-T at room temperature for 1 h. The plates were washed and incubated with 100 µl of Tetramethylbenzoate (Monobined, USA) for 30 min. The OD was measured in an ELISA reader at 450 nm (Anthos-2020, Austria). Forty serum samples, including 20 negative controls and 20 positive serums of the cases with *C. parvum* infection (confirmed by Ziehl-Neelsen staining) were analyzed to determine the specificity and sensitivity of the antibodies against rCP2 and rCP23 proteins. Furthermore, for cross-reactivity assessment with other parasites, positive serum for *Giardia lamblia*, *T. gondii*, *Entamoeba histolytica*,* Isospora belli*, hydatid cyst, and *Cyclospora cayetanensis *(n = 5 each) were used. The cut-off value was calculated as the mean OD ± three SD of negative control sera (Fig. 1).

**Bioinformatics analysis**

Sequence homology analyses were accomplished using the NCBI databases with the BLAST search tool (http://www.ncbi.nlm.nih.gov/). The Geneious software (version 4.8.5), a bioinformatics tool for annotating sequence alignment, was also used to compare the retrieved sequences.

**Statistical analysis**

The student’s *t*-test was used to evaluate the significant difference (*p *< 0.05) between OD values of known *C. parvum* positive and negative human sera in ELISA using GraphPad Prism version 4.0. Agreement between the molecular analysis (standard test) and rP23/rP2 ELISA test was estimated with the kappa coefficient (GraphPad software; http://graphpad.com/ quickcalcs/kappa1/).

## RESULTS

**Morphological identification of oocysts**

 The *Cryptosporidium* oocysts were isolated from the feces of HIV-positive patients by the sucrose gradient method. In Ziehl-Neelsen staining, *C. parvum* oocysts were observed as red spheres with the diameter of 2-6 microns.

**Codon** **optimization**

 Sequencing analysis of the amplified *P2* and *P23* gene revealed that these genes were 336 bp and 333 bp in length, respectively. The amino acid sequence encoded by these two synthesized genes were 100% consistent with those presented in the NCBI GenBank (accession numbers: AF099744 and U34390, respectively). The sequence of *P2* and *P23* genes were optimized based on *E. coli* codon bias *without changing* the *sequence* of the *amino acid. *

**Fig. 2:**
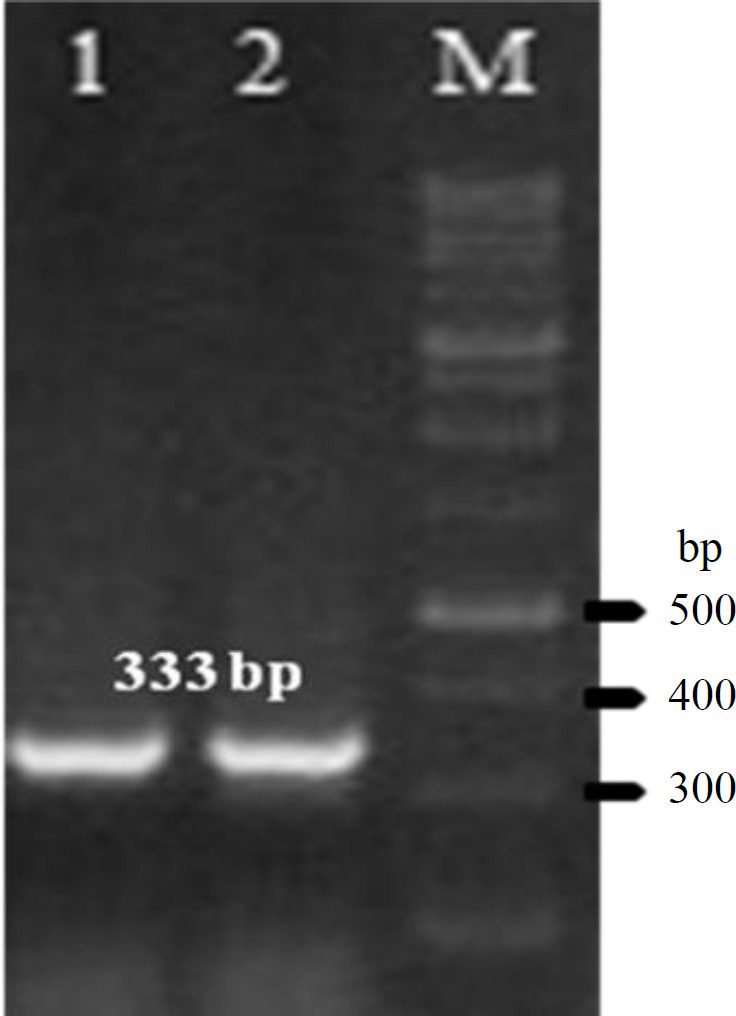
Confirmed cloning and transformation of recombinant expression plasmid in *E. coli BL21* (DE3) by PCR on 1% garose gel. Lane 1, *CP2*; lane 2, *P23*; M, DNA marker 100 bp

**Characterization of rP2 and rP23 proteins**

**Fig. 3:**
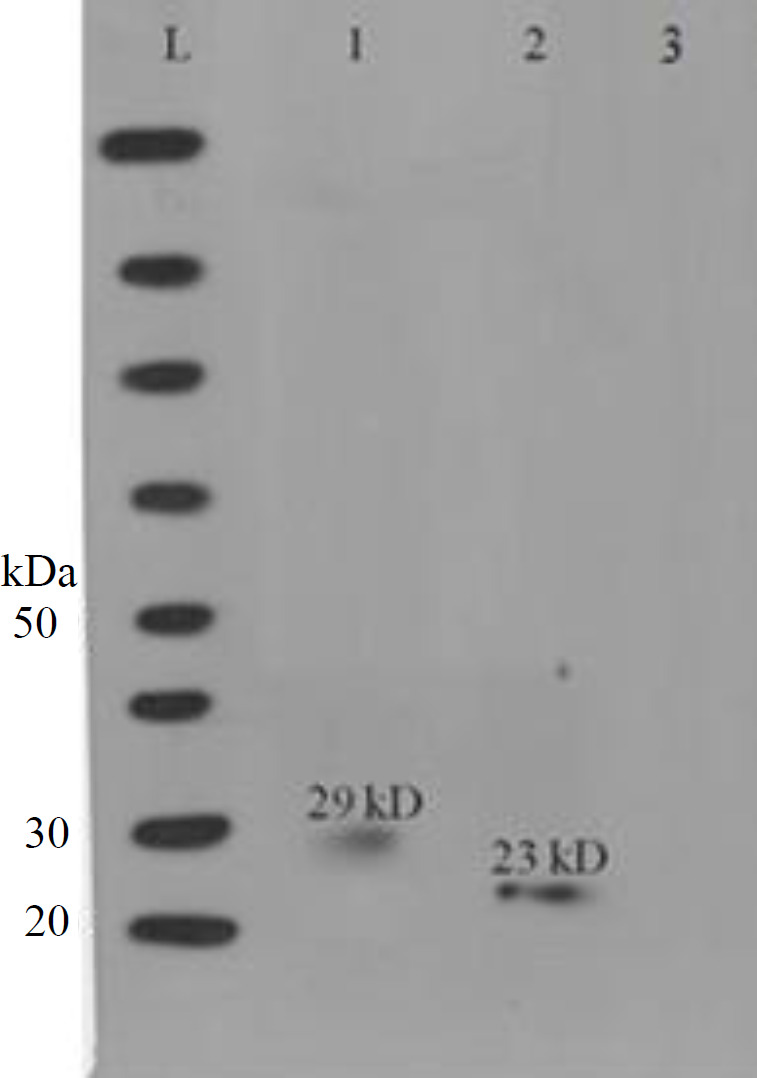
Result of Western blot test with serum at 1:200 dilutions. Lane L, protein marker; lanes 1 and 2, reaction of rP23 and rP2 antigens with serum antibodies, respectively; lane 3, total protein of untransformed *E. coli* BL21 in Western blot with the positive control serum pools

 The synthesized genes were cloned separately into plasmid pET28a+. Colony PCR was used to confirm the cloning of the genes (Fig. 2). The rP2/rP23 proteins were successfully expressed by* E. coli *BL21 DE3 strain under the conditions described in Materials and Methods. SDS-PAGE analysis of *E. coli *BL21 (DE3), which was transformed with *P23*-pET28a and *P2*-pET28a and induced with IPTG, showed the expected 29 kDa (*CP23* protein plus 6 kDa of His-tag) and 23 kDa (17 kDa *CP2* protein and 6 kDa of His-tag) bands, respectively. Western blotting results showed the detection of both rP2 and rP23 by specific *C. parvum* antibodies (Fig. 3). There was not any reaction between positive sera and proteins of untransformed BL21.

**Diagnostic potential of rP2/rP23 in ELISA**

**Fig. 4:**
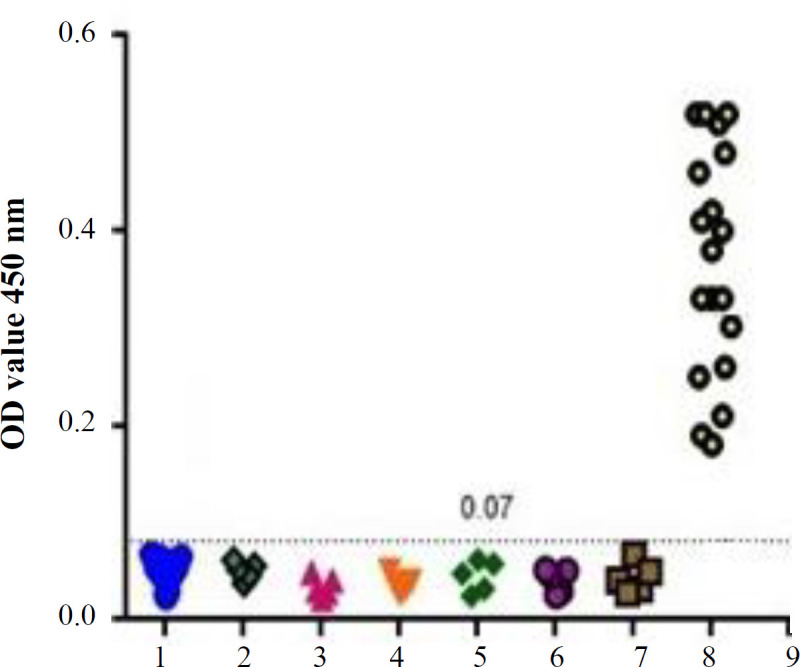
Crossreactivity of rCP23, rCP2 in ELISA method. 1, negative control (n = 20); 2, *Giardia lambellia* (n = 5); 3, *T. gondii* (n = 5); 4, *Entamoeba histolytica* (n = 5); 5, *Isospora belli* (n = 5); 6, *hydatid cyst* (n = 5); 7, *Cyclospora cayetanensis*; 8, *Cryptosporidium parvum*-infected human sera (n = 20)

For evaluation of the rP2/rP23 antigens as serodiagnostic candidates for C*. parvum* infection, a panel of sera, comprising of positive and negative sera and sera from human infected with other parasites, was used. The most appropriate concentration of recombinant antigens was 5 mg/well, and the most suitable dilution of the serum sample was 100 µ/well with 1: 100 dilutions for use in the ELISA method. Based on the results, the most appropriate dilution and amount of gout anti-human HRP-conjugated as secondary antibody was 100 µl of 1:5000 dilution. The cut-off value for negative sera was 0.07. The anti-rP2/rP23 antibodies were not observed in the control samples. Also, no cross-reaction was found between the recombinant antigens and positive human sera for *Giardia lamblia*,* T. gondii*, *Entamoeba histolytica*, *Isospora belli*, hydatid cyst, and *Cyclospora cayetanensi.* However, there was a significant difference between positive and negative cases in the presence of antibodies against *C. parvum *(*p *< 0.01; Fig. 4)*.*

## DISCUSSION

Evidence have shown that the simultaneous use of two different recombinant antigens could increase the sensitivity and specificity of the serodiagnosis^[^^16^^]^. Therefore, in the current study, the rP2/rP23 proteins were used for immunodiagnosis. 

The results of this study indicated that rP2/rP23 antigen has a high sensitivity and specificity for detecting specific antibodies against *C. parvum*. To better express the rP2 and rP23 in the bacteria, the genes encoding the desired proteins were optimized by JCat software and then chemically synthesized. In most studies, GST-Tag has been used to purify the expressed protein, which can increase the molecular weight of the expressed protein; however, this study used His-Tag to solve this problem. The purified proteins were much closer to natural proteins in terms of molecular weight. In previous studies, the purified rP23 had different molecular weights, such as 43 kDa^[^^13^^]^, 46 kDa^[^^17^^]^, 23 kDa^[^^18^^]^, 27 kDa^[^^1^^]^, 37 kDa^[^^11^^]^, and 40 kDa^[^^19^^]^, which may be due to the cloning system and expression methods. The type of Tags also affected the final molecular weight of the resulting protein. For instance, GST-Tag is much heavier than His-Tag. In the current study, His-tag sequences were added to the N-terminal part of *P2* and *P23* genes as this Tag do not affect protein structure and functions. After purification in SDS-PAGE, the molecular weights of rP2 and rP23 were 23 and 29 kDa, respectively.

The antigenicity of rP2/rP23 protein was evaluated and proved by Western blotting. In addition, rP2/rP23 ELISA test was able to detect the positive and negative human sera of *C. parvum* infection. In this study, no cross-reactivity with other parasite infections was detected. Moreover, no false-positive and false-negative cases were observed among the 20 positive and 20 negative sera, and they were 100% consistent with the standard test. According to these results, both sensitivity and specificity of rP2- and rP23-ELISA were 100%. In the study of Bannai *et al.*^[^^20^^]^, the values of sensitivity and specificity of the ELISA test for detecting cryptosporidiosis were 80% and 73.3%, respectively, which was lower those of our study. To the best of our knowledge, there is only one study concerning the evaluation of the recombinant antigens for detection of human cryptosporidiosis^[^^21^^]^, which had lower sensitivity and specificity than the current study.

The findings of this study reveal the high sensitivity and specificity of rP2-/rP23-ELISA for detecting specific antibodies against *C. parvum *infection. Furthermore, due to antigenic properties of rP2/rP23 antigen, it can be used for the diagnosis of human cryptosporidiosis.

## DECLARATIONS

### Acknowledgments

This study was financially supported by Shiraz University of Medical Sciences, Shiraz, Iran (Grant number: 21439). Authors would like to thank the personnel of the center of consultation and research of Shiraz University of Medical Sciences for the editorial assistance.

### Ethical statement

This study has been approved by the Ethics Committee of Shiraz University of Medical Sciences, Shira, Iran (Ethical code IR.SUMS.REC.1400.564).

### Data availability

The raw data supporting the conclusions of this article are available from the authors upon reasonable request.

### Conflict of interest

None declared.

### Funding/support

This study was financially supported by Shiraz University of Medical Sciences, Shiraz, Iran.
